# Volatile Compositional Profile, Antioxidant Properties, and Molecular Docking of Ethanolic Extracts from *Philodendron heleniae*

**DOI:** 10.3390/molecules30061366

**Published:** 2025-03-18

**Authors:** Melanie Ochoa-Ocampo, Nina Espinosa de los Monteros-Silva, Jefferson V. Pastuña-Fasso, Juan Diego Sacoto, María Cristina Peñuela-Mora, Gerardo Casanola-Martin, José R. Almeida, Karel Diéguez-Santana, Noroska G. S. Mogollón

**Affiliations:** 1 Biomolecules Discovery Group, Universidad Regional Amazónica Ikiam, Km7 Via Muyuna, Tena 150101, Ecuador; melanie.ochoa@ikiam.edu.ec (M.O.-O.); jefferson.pastuna@ikiam.edu.ec (J.V.P.-F.); juan.sacoto@est.ikiam.edu.ec (J.D.S.);; 2 Laboratorio de Biología Molecular y Bioquímica, Universidad Regional Amazónica Ikiam, Km7 Via Muyuna, Tena 150101, Ecuador; nina.espinosadelosmonteros@ikiam.edu.ec; 3 Ecosistemas Tropicales y Cambio Global, Universidad Regional Amazónica Ikiam, Km7 Via Muyuna, Tena 150101, Ecuador; 4 Department of Coatings and Polymeric Materials, North Dakota State University, Fargo, ND 58102, USA; gerardo.casanolamart@ndsu.edu; 5 School of Pharmacy, University of Reading, Reading RG6 6UB, UK

**Keywords:** gas chromatography, antioxidant, biodiversity, mass spectrometry, plant extracts, bioprospection, molecular docking

## Abstract

Antioxidants are essential compounds with diverse applications, and medicinal plants are a natural source of these biomolecules. *Philodendron heleniae*, a species native to the Ecuadorian Amazon, belongs to a genus renowned for its traditional therapeutic uses. Extracts from the stems and roots of several *Philodendron* species have been widely used to treat stress, bladder disorders, and snakebite wounds, underscoring their medicinal potential. This study investigates the volatile composition, antioxidant properties, and molecular docking of ethanolic extracts from *P. heleniae*, aiming to expand its applications. Phytochemical analysis revealed a rich profile of tannins, phenolic compounds, flavonoids, and terpenoids. Antioxidant assays (ABTS and DPPH) demonstrated the extract’s strong free radical scavenging capacity, comparable to the standard Trolox. GC-MS analysis identified 48 volatile and semi-volatile metabolites, predominantly phenolic compounds, terpenoids, and lipid-like molecules. Fractionation of the crude ethanolic extract into aqueous and ethanolic fractions simplifies the downstream analytical steps and facilitates the identification and the evaluation of the higher abundance of antioxidant-related metabolites. Molecular docking supported these findings, highlighting strong binding affinities of stigmasterol and desmosterol to catalase, an enzyme critical for reducing oxidative stress. These results position *P. heleniae* as a promising source of natural antioxidants with potential pharmaceutical applications, while emphasizing the importance of conserving Ecuador’s biodiversity and its bioactive resources.

## 1. Introduction

Antioxidants can be defined as substances that significantly delay or prevent substrate oxidation when present at low concentrations compared with an oxidizable substrate [1,2]. They are valuable in fields like medicine because it is well known that antioxidants protect against damage caused by free radicals, playing an important role in chronic diseases such as cardiovascular diseases, inflammation, cancer, anaemie, and aging [3,4]. Also, antioxidants display applications in the food industry, not only with the aim of enriching food but also for developing edible films and packaging materials for food, improving oxidation stability, and prolonging shelf life [3,5,6,7]. In a different area of study, antioxidant molecules are commonly added to products such as stabilizers in fuels and lubricants to prevent oxidation, and in gasoline to prevent polymerization [8].

Antioxidants are usually synthetic; however, due to factors such as their high volatility and instability at elevated temperatures, currently, the interest in using natural antioxidants has increased because they represent less costs, and, in some cases, they demonstrate better activity [2,3,9,10]. They are commonly present in plants, placing them as a basic source, where many antioxidants have been identified as free radicals or active oxygen scavengers [11,12]. In particular, medicinal plants have reported great antioxidant potential, and some authors have indicated approximately two-thirds of all plant species have medicinal potential [13,14].

In this context, we can mention Ecuador, one of seventeen megadiverse countries on Earth that possesses about 10% of all plant species [15,16,17]. Moreover, its Amazon region represents one of the largest ecological reserves due to its great diversity in flora and fauna and the ecosystem services it provides [15]. However, despite the ethnopharmacological information and traditional knowledge of medicinal plants from there, some are still not chemically characterized or evaluated for antioxidant potential. It is important not only to increase data about plant content with an industrial aim but also to validate the empirical knowledge of communities.

Related to species with promising applications, we found *Philodendrum heleniae,* belonging to the *Philodendron* genus and Araceae family [18,19]. It is native to Central America and is distributed in tropical humid and rainy forest habitats of Panama, Ecuador, and Colombia [20]. In Ecuador, it is mainly found in the Amazon region [21,22]. In the Ecuadorian Amazon Region, the stems and root extracts of the *Philodendron* genus have been reported as a treatment for stress, bladder problems, and ophidian accidents, as well as being used as a cicatrizant and antihemorrhagic [23,24,25,26]. Meanwhile, experimental reports of species belonging to this group, such as *P. megalophyllum, P. erubescens,* and *P. bippinatifidum,* revealed promising antimicrobial and antioxidant activity [27,28]. These activities are due to the variety in the chemical composition that these plants possess, which includes a variety of sesquiterpenes (E-β-farnesene, germacrene-D, β-caryophyllene, trans-α-bergamotene) [29,30], flavonoids (luteolin and quercetin), phytosterols (β-sitosterol and stigmasterol) [31], 5-alkyl and 5-alkenylresorcinols (allergens), fatty acid ethyl esters, a polyprenoid (hexapreol), and aromatic amines [32].

Regardless of the potential of the *Philodendron* genus, *P. heleniae* has not been deeply studied yet. Hence, in this work, we want to evaluate its antioxidant activity and identify the potential molecules closely related to it, searching to expand the applications of this interesting species. This work contributes to promoting the broad study of biological resources, helping to highlight all their potential uses and also remarking on the importance of their conservation.

## 2. Results

### 2.1. Phytochemical Screening

Phytochemical analysis of the crude extracts was carried out to identify potential bioactive compounds (Table 1). The qualitative results revealed the presence of tannins, phenolic compounds, quinones, and flavonoids.

### 2.2. Total Phenolics and Flavonoids Content

The quantitative colorimetric analysis confirmed a significative content of polyphenols and flavonoids (Table 2).

### 2.3. Antioxidant Assay

The results of antioxidant activity evaluated using the 2,2′-azino-bis(3-ethylbenzothiazoline-6-sulfonic acid) (ABTS) and 2,2-diphenyl-1-picrylhydrazyl (DPPH) methodologies are shown in Table 3. It is expressed as the equivalent antioxidant capacity of the standard Trolox (mg of Trolox equivalents/grams of dry weight). The data are represented as the mean ± SD of three measurements.

### 2.4. GC-MS Analysis

**Figure 1:**
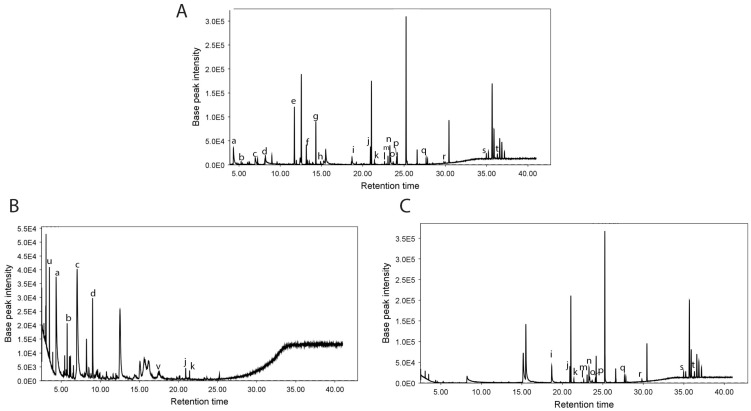
Chromatographic profiles of the extracts obtained from *P. heleniae* by gas chromatography coupled to mass spectrometry (GC-MS): (**A**) crude extract, (**B**) aqueous extract, and (**C**) ethanolic extract. The peaks represent the identified compounds, highlighting the following: a = Propiophenone, b = 3,7-Dimethyltropolone, c = Methyl 3-hydroxyhexadecanoate, d = 2,3,5-Trimethyldecane, e = Alpha-Cubebene, f = 1-Pentadecanol, g = 5-Azulenemethanol, h = Trans-Nerolidyl formate, i = Protocatechuic acid methyl ester, j = Octadecanoic acid, k = Octadecanoic acid, ethyl ester, l = Muscalure, m = Isophytol, n = Trans-9, Trans-12-Octadecadienoic Acid Methyl Ester, o = (11Z,14Z,17Z)-Methyl icosa-11,14,17-trienoate, p = Hexadeca-2,6,10,14-tetraen-1-ol, 3,7,11,16-tetramethyl, q = 1,3-Propanediol, eicosyl ethyl ether, r = Desmosterol, s = Fumaric acid, dec-4-enyl hexadecyl ester, t = Stigmasterol, u = Isofucosterol, v = Cyclohexanol.

GC-MS analysis was conducted to investigate the volatile and semi-volatile metabolites in the ethanolic extracts *P. heleniae* (Figure 1). A total of 48 compounds were tentatively identified. Among these, 30 components were classified as level 2 identifications using the NIST 20 EI library, MS-DIAL library (https://systemsomicslab.github.io/compms/msdial/main.html, accessed on 5 September 2024), and GNPS, based on spectral similarity, and the Van den Dool and Kratz linear retention index (Table 4). The remaining 21 components were identified at level 3, prioritizing their fragments and molecular mass.

**Figure 2:**
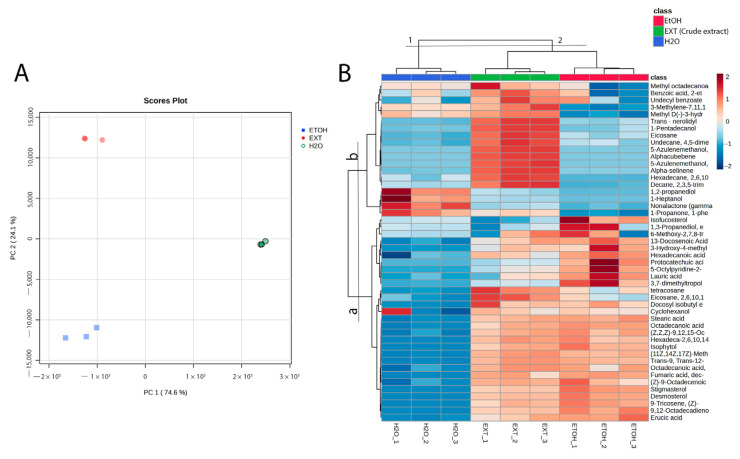
(**A**) Principal component analysis (PCA). (**B**) Heatmap and HCA; cold colors (blue scale) represent a low metabolite abundance, while warmer colors (red scale) indicate a higher abundance.

The variability of components in both fractions (aqueous and ethanolic), aimed at identifying those with a higher likelihood of exhibiting antioxidant activity, was evaluated using principal component analysis (PCA), heatmap visualization, and hierarchical cluster analysis (HCA) (Figure 2).

The PCA score plot revealed two clusters along PC1, which explained 74.6% of the variance (Figure 2A). The first cluster, located in the negative PC1 region, included the crude extract and ethanolic fraction, while the second cluster, positioned in the positive PC2 region, contained the aqueous extract. Related to sample composition, a notable variation was observed in the relative abundance of the identified compounds. The majority of components, such as phenolic compounds, terpenoids, lipids, and lipid-like molecules, were found in higher concentrations in the ethanolic fraction. Conversely, certain benzenoids and organic oxygen compounds, which are not directly associated with antioxidant activity, were predominantly present in the aqueous fraction. According to the hierarchical cluster analysis shown at the top of the heatmap (Figure 2B), group 1 consists of components primarily found in the aqueous fraction, which also showed the lowest relative abundance of compounds. On the other hand, group 2, which includes the crude extract and ethanolic fraction, displayed the same trend observed in the PCA, with these samples containing the metabolites in higher abundance.

**Figure 3:**
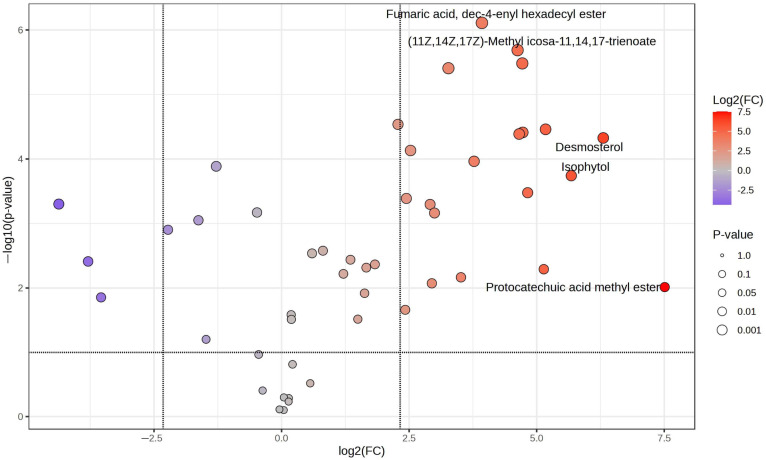
Volcano plot showing differential metabolite abundance in both fractions: blue for aqueous and red for ethanolic fraction.

With the primary goal of visualizing the abundance of components in the ethanolic fraction while avoiding the complexities of the crude extract that may obscure identification, we applied the Volcano plot with fold change (FC) > 5.0 and *p*-value < 0.05 between the fractions obtained (Figure 3). This approach enabled us to highlight components with higher statistical significance by contrasting their presence in the ethanolic fraction and their absence or low presence in the aqueous fraction.

The volcano plot visualization identifies metabolites with significant relative abundance differences, highlighting those more likely associated with antioxidant activity due to their higher affinity for the ethanolic fraction, emphasizing fumaric acid, dec-4-enyl hexadecyl ester, (11Z,14Z,17Z)-methyl icosa-11,14,17-trienoate, desmosterol, isophytol, protocatechuic acid methyl ester, 3-Hydroxy-4-methylbenzoic acid, (Z)-9-Octadecenoic acid methyl ester, erucic acid, and stigmasterol.

### 2.5. Molecular Docking

**Figure 4:**
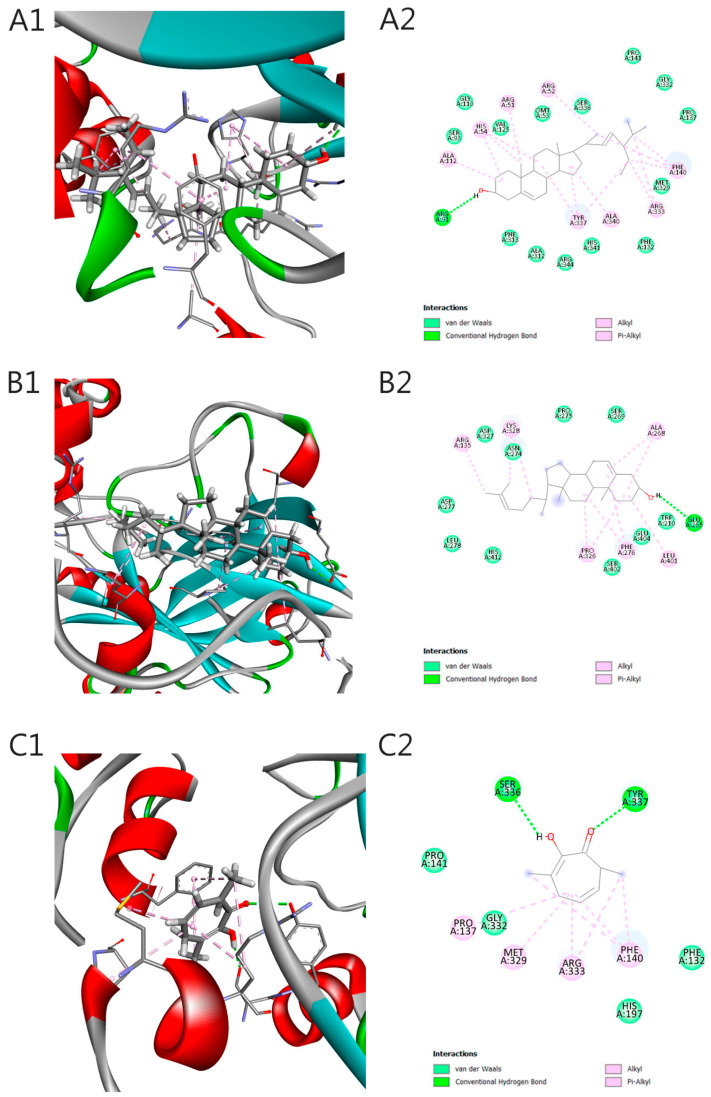

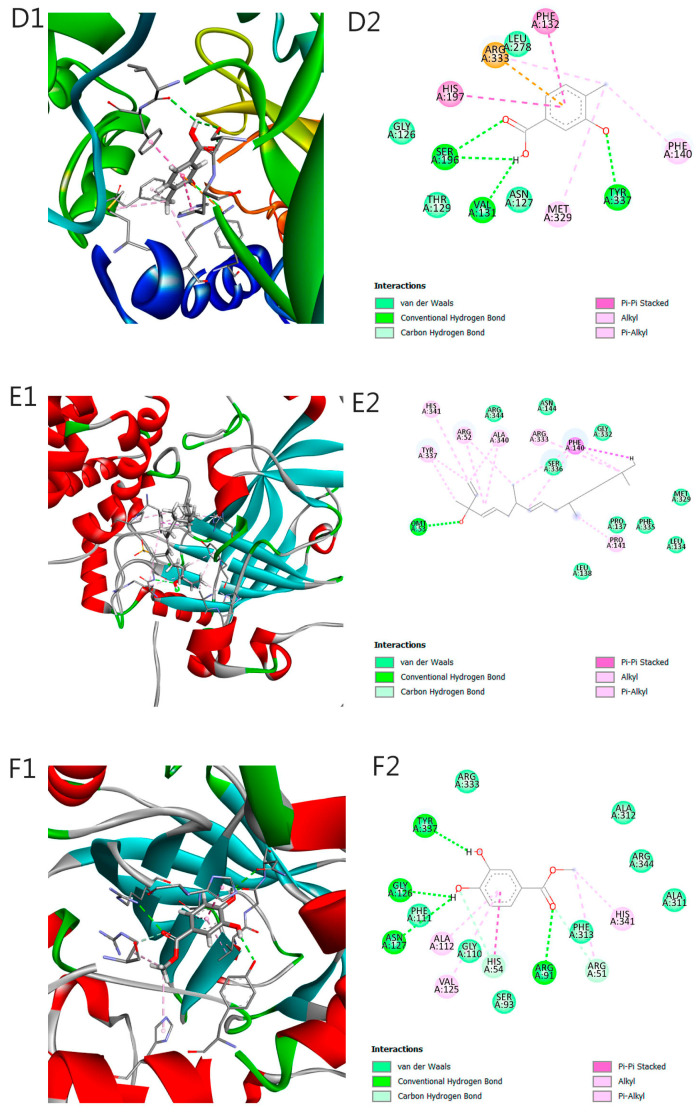
Visualization of interaction between protein 2CAG and ligands isolated of *P. heleniae*. (3D and 2D interactions). (**A**) Stigmasterol. (**B**) Desmosterol. (**C**) 3,7-dimethyltropolone. (**D**) 3-Hydroxy-4-methylbenzoic acid. (**E**) Isophytol. (**F**) Protocatechuic acid methyl ester.

Molecular docking analysis of compounds present in the ethanolic extract of *P. heleniae* against catalase (PDB ID: 2CAG) identified ligands with significant affinities towards the active site of the enzyme, with stigmasterol and desmosterol standing out as the most promising compounds. Stigmasterol presented the lowest binding energy (−11.0 kcal/mol) and an inhibition constant (Ki) in the nanomolar range (8.58 nM), indicating a high ability to stabilize at the active site (Table 5). This compound interacts via hydrogen bonding with Arg91, a key residue in the catalytic function of catalase, complemented by 10 Van der Waals interactions, including Asp327, His412, and Trp210, in addition to hydrophobic contacts with Pro326, Leu401, and Phe276 (Figure 4A1,A2). These interactions reinforce the orientation and stability of the complex, evidencing efficient coupling and specificity towards the enzyme.

Regarding this, desmosterol showed a slightly lower affinity energy (−8.3 kcal/mol; Ki = 0.82 µM), stabilizing through hydrogen bonding with Glu265 and a significant number of hydrophobic and pi-alkyl interactions with residues such as Ala312, His54, and Tyr337 (Figure 4B1,B2). These interactions highlight its potential as an effective catalase modulator, referring to stigmasterol’s ability to stabilize the enzyme’s structure or prevent its inactivation, rather than directly accelerating its enzymatic activity. This stabilization could help reduce oxidative stress by maintaining catalase function in the presence of ROS. Other compounds evaluated, such as 3,7-dimethyltropolone, 3-hydroxy-4-methylbenzoic acid, isophytol, and protocatechuic acid methyl ester, showed moderate affinities, with binding energies between −7.0 and −6.0 kcal/mol. Although these affinities are lower, the ligands formed specific interactions, such as hydrogen bonds with Tyr337 and Ser196 (Figure 4, Ligand C, D and F), and hydrophobic interactions with Met329 and Pro137 (Pi-alkyl, Figure 4, C and E), which could be exploited to optimize their chemical structures in future studies.

The diversity of molecular interactions observed evidences the flexibility of the catalytic site of catalase to accommodate different types of ligands, including steroids, terpenoids, and phenolic compounds. Correlations between binding energies and inhibition constants highlight stigmasterol and desmosterol as prime candidates for further investigations. However, while stigmasterol binds near the heme region, this does not necessarily indicate inhibition; it may also contribute to enzyme stabilization or modulate its catalytic activity. Further experimental validation is needed to clarify the precise impact of this interaction. Residues such as Arg91, Tyr337, and Glu265 are not only involved in ligand stabilization (Figure 4. Ligand C, D and F), but are also related to the catalytic function of the enzyme, highlighting the importance of these interactions in the context of modulating its activity. The relevance of these findings is that the tested compounds, as part of the ethanolic extract of *P. heleniae*, could act as natural antioxidants by stabilizing catalase activity, protecting cells against oxidative damage and reducing reactive oxygen species (ROS) levels.

Additionally, we compared the three-dimensional structures of 2CAG and human erythrocyte catalase (8HID), using PyMOL to assess their structural conservation and identify functional differences. Using the align command, we superimposed the structures, optimizing the fit by refinement cycles that rejected mismatched atoms. The overall RMSD was 0.70 Å for 2709 aligned atoms, and the active site RMSD was 0.43 Å, indicating high structural similarity, especially in the active site, where key residues and the heme group are nearly identical. The alignment converged in a single cycle, confirming the similarity between the structures. The differences were located in peripheral regions or flexible loops, less critical for catalytic activity. These results support the relevance of 2CAG as a model for functional and ligand interaction studies in human catalase (See Figure S3 and Figure S4 of the Supplementary Material).

While our primary focus was not antimicrobial, future studies will evaluate cross-reactivity with human catalase (PDB: 8HID) to ensure therapeutic safety. Catalase active sites are highly conserved across species, supporting the use of 2CAG as a model to study relevant interactions in human catalase.

## 3. Discussion

In the current phytochemical screening of the ethanolic extract of *P. heleniae*, a predominant presence of phenolic compounds and tannins was found (Table 1). This is the first study to report the presence of key metabolites in the ethanolic extract of this species. However, the results coincide with previous research on plants of the same genus that offer important insights. For example, the phytochemical analysis of *P. megalophyllum* highlights the presence of tannins, which are often recognized as key contributors to coagulant, edematogenic, and hemorrhagic activities [24]. Similarly, *Philodendron erubescens* ’Imperial Red’ has been documented to contain tannins, as well as triterpenoids and flavonoids [31]. These bioactive compounds are widely recognized for their medicinal benefits and have shown potential therapeutic applications in treating various diseases [32,33,34,35,36].

The results obtained in this study reveal a concentration of 574.7 µg/mg (574.7 mg/g_extract_) for total phenols and 2.5 µg/mg (2.5 mg/g_extract_) for total flavonoids in the analyzed hydroethanolic extract. When comparing these values with those reported in the literature, the study by [24] on *P. megalophyllum* reports 13.96 g/100 g (139.6 mg/g_extract_) for total phenols and 1.63 g/100 g (16.3 mg/g_extract_) for total flavonoids in an aqueous extract. While our phenolic content is significantly higher than that reported, the flavonoid concentration in our study is markedly lower. These differences could be attributed to variations in plant species, the solvent system used, and the extraction methodology, as phenolic compounds exhibit different solubilities depending on the polarity of the solvent. Additionally, the study by [37] on *P. adamantinum* quantifies 52.43 mg/g of total flavonoids in a hydroethanolic extract, a much higher value than the 2.5 mg/g found in this study. The variability in metabolite concentrations may result from species-specific metabolic profiles or environmental factors affecting compound biosynthesis [37]. It is important to highlight that there are no previous studies quantifying phenols and flavonoids in crude extracts of other *Philodendron* species, making the results obtained in this study a valuable contribution to the phytochemical characterization of the genus.

Considering the high concentration of phenolic compounds and their well-documented ability to neutralize free radicals by donating electrons or hydrogen atoms [37], the antioxidant potential of the crude extract was assessed using both ABTS and DPPH assays. In the ABTS assay, the extract exhibited a value of 1.03 ± 0.02 TEAC, closely mirroring the performance of the standard antioxidant Trolox, as indicated by the similar EC50 values (58.36 mg/L for the extract versus 59.80 mg/L for Trolox) (Figure S1). These results highlight the broad-spectrum effectiveness of the phenolic-rich extract, considering that the ABTS assay measures the scavenging capacity of both hydrophilic and lipophilic antioxidants [38]. Conversely, while the DPPH assay also confirmed antioxidant activity (0.67 ± 0.06 TEAC), its EC50 (20.53 mg/L) differed more markedly from Trolox (14.65 mg/L) (Figure S2). The EC50 value obtained is very similar to that reported in another species, where for the DPPH test the aqueous extract of *P. megalophyllum* had an EC50 of 22.5 ± 1.78 mg/L [24]. Furthermore, this study [24] suggests that the observed activity may be linked to the presence of phenolic compounds, which have the ability to scavenge free radicals or chelate metal ions, thereby preventing oxidation. This finding is consistent with the high concentrations of phenolic compounds obtained in this study, which likely contribute to the extract’s remarkable antioxidant activity. Taken together, these findings underscore the significant contribution of phenolic compounds to the extract’s bioactive potential.

To achieve a clearer understanding of the chemical complexity of the extract and to establish more precise correlations between specific metabolite groups and their biological activities, a targeted fractionation approach was employed. By dividing the crude ethanolic extraction into aqueous and ethanolic fractions, it not only simplifies the downstream analytical steps, but also facilitates the identification and the evaluation of the relative abundance of distinct types of bioactive compounds (Figure 1). It improves the detection of subtle or low-abundance metabolites by eliminating the matricial effect. This is related to the intrinsic polarity differences, as more polar compounds remain in the aqueous fraction, while less polar metabolites are concentrated in the ethanolic fraction [39,40,41].

Taking advantage of this fractionation, antioxidant compounds that are usually lipophilic or semipolar are expected to be concentrated predominantly in the ethanolic fraction rather than the aqueous one [41,42]. To identify the metabolites obtained from fractionation, gas chromatography–mass spectrometry (GC-MS) was employed, enabling precise identification. We identified 48 compounds in the ethanolic extract, among which phenolic compounds, terpenoids, lipids, and lipid-like molecules were predominant. Notably, metabolites such as stigmasterol, protocatechuic acid methyl ester, (Z)-9-octadecenoic acid methyl ester, and desmosterol stood out due to their known antioxidant properties. Further research on this genus has primarily focused on the chemical composition of essential oils extracted from the roots of various *Philodendron* species [28,43,44,45]. These studies have identified mainly sesquiterpenes as the most diverse and abundant compounds, together with the presence of some flavonoids and phytosterols [27,45,46].

This study identified a broader chemical diversity compared to previous reports on plants of the same genus. A possible explanation for this chemical diversity could be attributed to differences in the species studied, the source material, which in most studies consists of essential oils, the type of solvents used, or the unique geographical and ecological conditions of the Ecuadorian Amazon. This is because factors such as climate, soil composition, altitude, and interactions with other organisms are known to significantly influence the metabolic profile [47].

The principal component analysis (PCA) revealed a clear separation of the ethanolic and aqueous fractions, with the ethanolic fraction clustering in regions associated with a higher abundance of metabolites linked to antioxidant activity (Figure 2A). The hierarchical cluster analysis (HCA) further corroborated these results, grouping the ethanolic extract distinctly from the aqueous one based on the metabolite profiles (Figure 2B). Additionally, the volcano plot highlighted statistically significant metabolites, such as fumaric acid, dec-4-enyl hexadecyl ester, (11Z,14Z,17Z)-methyl icosa-11,14,17-trienoate, desmosterol, isophytol, protocatechuic acid methyl ester, 3-Hydroxy-4-methylbenzoic acid, (Z)-9-Octadecenoic acid methyl ester, erucic acid, and stigmasterol (Figure 3), which are more likely to contribute to the strong antioxidant activity observed in the assays because of their relation and higher relative abundance in this fraction. This metabolomic approach not only confirmed the relative abundance of bioactive compounds in the ethanolic crude extract but also provided insights into specific metabolites that may drive the observed bioactivity.

Molecular docking was conducted on all molecules with the highest statistical significance (Figure 2); however, only those with the best results are presented. This analysis aimed to reinforce our findings by demonstrating that certain identified compounds exhibit affinity for catalase. The enzymatic antioxidant catalase (CAT) was selected as a target for the antioxidant activity of phytochemicals. The main function of this enzyme is to decompose hydrogen peroxide into water and oxygen (neutralizing reactive oxygen species). Therefore, it plays an important role in cellular protection against oxidative stress by interacting with other compounds with antioxidant properties [48,49].

The results obtained in the molecular docking study between compounds from *P. heleniae* and catalase (PDB ID: 2CAG) find support in previous research that has explored similar interactions between plant extracts and antioxidant enzymes. For example, a study on the plant *Labisia pumila* (Kacip Fatimah) analyzed the interaction between secondary metabolite ligands and the target antioxidant protein (catalase (2CAG)) to find the binding rate between the constituent molecules [50]. They found that flavonoids demonstrated superior antioxidant activity (lower energy interaction in scoring function) and confirmed significant antioxidant activity through DPPH scavenging. Similarly, [51] studied the interactions of phytochemicals from six local Indian plants (pomegranate, lemon, wheatgrass, papaya, sheesham leaves, turmeric leaves) against the 2CAG enzyme. Their results showed that several compounds had good affinity for catalase.

The calculated Ki values (nM–μM range) suggest biologically relevant binding affinities, highlighting stigmasterol and desmosterol as promising candidates for future investigations. Although our docking results indicate that stigmasterol binds close to the heme region, this does not necessarily imply a direct increase in catalytic activity but rather an indirect stabilization that could favor its function under oxidative stress. However, the precise mechanism of this modulation requires additional experimental validation, such as kinetic assays and molecular dynamics simulations. Since catalase is already one of the fastest known enzymes, alternative approaches such as overexpression or modulation of its expression could be explored in future studies to enhance cellular antioxidant defenses. Furthermore, these findings are congruent with the interactions observed between *P. heleniae* compounds and catalase, where ligands such as stigmasterol and desmosterol showed favorable binding energies and multiple stabilizing interactions with key residues of the enzyme. For example, stigmasterol has demonstrated significant antioxidant activities. In their work, [52] indicate that this compound can reduce lipid peroxidation and DNA damage, conferring chemoprotective properties in skin cancer [53]; stigmasterol attenuates excitotoxicity, DNA damage, and mitochondrial dysfunction and decreases ROS production. Furthermore, [54] investigated the protective activity of ethanol extracts of *Grewia carpinifolia* against vanadium-induced toxicity in mice, identifying β-sitosterol and stigmasterol as the primary active components of the extracts.

While our study focused on the heme-active site due to its critical role in catalase’s catalytic function, we acknowledge that catalase, as a tetramer, may harbor additional ligand-binding sites, including regions relevant to NADPH binding. Future studies could explore these sites to provide a more comprehensive understanding of potential inhibitory mechanisms. Additionally, the protonation states of residues were assigned at pH 7.4, and further refinement using molecular dynamics simulations to account for environmental pH variations could enhance the accuracy of our findings. These results demonstrate that the integration of enzymatic tools, chemical profiling, and in silico analysis provided a comprehensive approach to identify the antioxidant potential of *P. heleniae*. This study lays the foundation for further exploration of *P. heleniae* as a natural source of antioxidants with potential pharmacological applications. Future research should focus on optimizing extraction techniques to enhance the yield and concentration of these bioactive compounds, while ensuring their stability and bioavailability. Additionally, efforts should be made to isolate and characterize individual metabolites, as well as examine their synergistic effects, to validate their specific biological activity and explore their therapeutic potential in in vivo models. Investigating the ecological and chemical variability of *P. heleniae* under varying environmental conditions could also offer valuable insights to maximize its bioactive properties for practical applications.

## 4. Materials and Methods

### 4.1. Plant Material

*Philodendron heleniae* was collected near the Universidad Regional Amazónica Ikiam (0°57′20.89″ S, 77°40′24.63″ W), Napo, Ecuador. The botanical sample was deposited at the Herbario Nacional del Ecuador (QCNE) with voucher QCNE-030-2021. Since local communities use this species in traditional medicine, we limited our sample collection to three replicates to avoid impacting the availability of this resource. The plants were carefully extracted from their native substrate and placed on dry ice to quickly quench metabolism. Subsequently, the tubers were transported to the laboratory and stored at −80 °C for further analysis.

### 4.2. Preparation of P. heleniae Crude Extracts

Powdered roots were macerated in ethanol (purity ≥ 99.8%) purchased from Sigma-Aldrich (Darmstadt, Germany) in a relationship of 1:10 (m/v) for 72 h, shaken every 24 h at room temperature. The resulting extract was filtered through 15 μm cellulose filter paper (MicroLAB Scientific, Yueqing, China) and concentrated using a rotary evaporator (Buchi, R-300, Flawil, Switzerland). Subsequently, the extract was dried using a vacuum dryer (Geneva, Mi Vac Duo, Warminster, PA, USA). Finally, it was stored at −20 °C until further use.

### 4.3. Phytochemical Analysis

The ethanolic extracts were subjected to phytochemical profile profiling using standardized methods [55,56] with some modifications. The qualitative evaluation of flavonoids, terpenoids, alkaloids, tannins, and quinones was carried out through colorimetric and precipitation reactions, as described in the referenced methodologies. The main modifications included the following: a 5 mg/mL solvent ratio was used, ultrasonic mixing was used for 5 min when preparing the samples, the concentration of ferric chloride was increased by 5%, and incubation times were standardized at 3 min for data collection. The qualitative assessment was classified as strongly positive (+++), positive (++), weakly positive (+), and not detected (−).

### 4.4. Total Phenol Content

The phenolic content of each extract was analyzed with the Folin–Ciocalteu (Supelco^®^) colorimetric method [57], with gallic acid as the standard. A 1 mg/mL (prepared by dissolving 5 mg of extract in 5 mL of ethanol) quantity was mixed with 860 μL of water and 40 μL of Folin–Ciocalteu in a light-protected environment and allowed to react for 5 min. Following this, 100 μL of a 7% Na_2_CO_3_ solution was added, and the mixture was left to stand for 60 min. The absorbance of the resulting mixture was measured at 750 nm using a Shimadzu UV-1280 spectrophotometer (Shimadzu Corporation, Kyoto, Japan). A calibration curve of gallic acid (ranging from 25 to 500 μg/mL) was used as a reference standard to calculate the phenolic content. The total phenolic content of the extracts was expressed as μg of gallic acid equivalent per mg of extract.

### 4.5. Total Flavonoid Content

The total content of flavonoid was determined using the aluminum trichloride (AlCl_3_) method [58], with quercetin as the standard. A 1 mL sample (4 mg of ethanolic extract dissolved in 4 mL of ethanol), was mixed with 1 mL of 2% AlCl_3_ and incubated at room temperature for 10 min The absorbance was measured at 438 nm using a Shidmazu UV-1280 spectrophotometer. A calibration curve was prepared within the range of 1–15 μg/mL, and the results were expressed as µg of quercetin per mg of extract.

### 4.6. Antioxidant Activity Evaluation

In this study, two different scavenging assays were used to determine the antioxidant activity from ethanolic extract of *P. heleniae*; DPPH (2,2-diphenyl-1-picrylhydrazyl radical) and ABTS (2,2′-azino-bis(3-ethylbenzothiazoline-6-sulfonic acid) radical) assays were used with the crude extract.

#### 4.6.1. ABTS Radical Scavenging Assay

The ABTS assay was estimated using the method of [59], although with some modifications. Firstly, a stock solution of ABTS was prepared by dissolving 7 mM of ABTS in distilled water and activating it with 2.45 mM potassium persulfate. The solution was incubated in the dark at room temperature for 16 h to generate the ABTS⁺ radical cation. The working solution was obtained by diluting the activated ABTS⁺ solution with ethanol until an absorbance of 0.70 ± 0.02 at 734 nm was achieved using a spectrophotometer (Shimadzu 3600 Plus, Kyoto, Japan).

Samples were prepared by dissolving them in ethanol and being sonicated for 5 min. For the assay, 150 μL of extract samples or standard solutions of trolox (6-hydroxy-2,5,7,8-tetramethylchroman-2-carboxylic acid) prepared at varying concentrations was added to 2850 μL of the free radical solution. The absorbance was measured at 734 nm after 6 min incubation at room temperature. The Trolox standard solution was used to construct the calibration curves, and the results are expressed as mg Trolox equivalents/grams of dry weight; the results are an average of three independent measurements. The ABTS activity was expressed as a percentage of inhibition and calculated using Equation (1).

(1) $$\text{ABTS}\;\text{radical}\;\text{scavenging}\;\text{activity}\left(\%\right)=\frac{(\text{Abs}\;\text{control}-\text{Abs}\;\text{sample})\times 100}{\text{Abs}\;\text{sample}}$$

where Abs control and Abs sample correspond to the absorbance of the blanks and the sample, respectively.

#### 4.6.2. DPPH Radical Scavenging Assay

The assay of DPPH was carried out according to the method outlined by [60], with slight modifications. The assay was performed in transparent 96-well plates, where 100 μL of ethanolic extracts at different concentrations and 100 μL of 96% ethanol were added to each well. Following this, 50 μL of DPPH solution (0.06 mM in 96% ethanol) was introduced. The plates were then incubated in the dark for 30 min under constant agitation at 60 rpm using an orbital shaker (Orbit™ LS Low Speed Laboratory Shaker, Labnet, Edison, NJ, USA) at room temperature. Absorbance measurements were taken in triplicate at 517 nm using a microplate reader (GloMax^®^, Promega Corporation, Madison, WI, USA). Calibration curves were constructed using a Trolox standard solution, and the results were expressed as milligrams of Trolox Equivalent Antioxidant Capacity (TEAC). Each experiment was carried out in triplicate.

### 4.7. GC-MS Characterization

#### 4.7.1. Sample Preparation

A total of 10 mg of the samples was dissolved in 1 mL of ethanol and vortexed for 1 min. Subsequently, 5 mL of the sample solution was filtered and fractionated using a cartridge containing 600 mg of silica gel (Sigma-Aldrich, Darmstadt, Germany). The cartridge was preconditioned with 15 mL of ultrapure water followed by 6 mL of ethanol. For the elution, 10 mL of ultrapure water was used to obtain the aqueous fraction, followed by 6 mL of ethanol to recover the ethanolic fraction. All the fractions were collected and dried using a dryer (Thermo Fisher Scientific, Waltham, MA, USA). Finally, the aqueous and ethanolic fractions were resuspended in 1 mL of ethanol. Metabolomic analysis was then performed by injecting the fractions and the crude ethanolic extract. For the GC-MS analysis, 150 μL of the upper transparent layer was mixed with 20 μL of methanol and 30 μL of caffeine (1.5 mg/L) as an internal standard; also, a process blank, solvent blank, and pooled QCs were included for GC-MS analysis to ensure the accuracy of the analysis.

#### 4.7.2. Metabolomic Data Acquisition and Metabolite Profiling

The chemical composition of the samples was analyzed using an AOC-6000 autosampler (Shimadzu Corporation, Kyoto, Japan) coupled to a GCMS-QP2020 NX quadrupole mass spectrometer (Shimadzu Co.) with electron ionization (EI). The system was equipped with an Rtx-5MS capillary column (30 m × 0.25 mm i.d. × 0.25 μm film thickness) coated with a 5% diphenyl/95% dimethylpolysiloxane stationary phase. A 1.0 μL aliquot of the sample was injected into the GC-MS system. The oven temperature was programmed as follows: an initial temperature of 70 °C was ramped at 6 °C/min to a final temperature of 300 °C, where it was held for 10 min, resulting in a total analysis time of 47.45 min. Ultrapure helium served as the carrier gas, with a constant flow rate of 1 mL/min. The injection port and transfer line temperatures were maintained at 200 °C and 220 °C, respectively. The ion source temperature was set at 200 °C, with electron ionization at 70 eV. The mass spectrometer scanned ions in the range of *m/z* 50 to 500 Da.

The metabolomics feature identification was performed using data from Shimadzu (.qdf) files, which were converted to (.mzML) format via ProteoWizard. Spectral data were deconvoluted in MS-DIAL version 4.9.221218 (http://prime.psc.riken.jp/, accessed on 1 June 2024), following a workflow that included peak detection, alignment, gap filing, and blank filtering, where the maximum sample intensity to average blank intensity ratio exceeded 7. The resulting feature list (*txt) from MS-DIAL was imported into the notame R package for data preprocessing, including normalization and drift correction. Upon completion of these steps, the proceeded data were then exported to MetaboAnalyst 6.0 for further statistical analysis.

### 4.8. Computational Molecular Docking Analysis

The crystal structure of the antioxidant protein was obtained from the PDB protein data bank (https://www.rcsb.org/, accessed on 2 November 2024), (PDBID: 2CAG). The selection of Proteus mirabilis catalase (2CAG) was based on its well-resolved structure and significance in oxidative stress models. Notably, this structure was chosen for its high resolution (2.7 Å) and the absence of co-crystallized ligands, which facilitates an unbiased exploration of potential binding sites. Protonation states were assigned using AutoDock Tools at pH 7.4, though future studies could consider environmental pH variations (e.g., lysosomal vs. cytoplasmic) for further refinement. The ligand (except for the heme group) and water atoms were removed, while the nonpolar hydrogens were fused. The protein structure was minimized and optimized using AutoDock Tools (ADT), accessed on 2 November 2024 included in the MGLTools package (version 1.5.7), to add charges and polar hydrogen atoms [61,62]. Ligand flexibility was considered by defining rotatable bonds, while the protein structure was kept rigid during docking. The active binding site of the catalase was chosen as the center of the grid, where the heme group and active site are located. The dimensions of the central grid box were chosen to include all atoms in the ligand pool. The grid box dimensions were set to 24.375 Å × 24.375 Å × 24.375 Å, centered at coordinates (63.366, 18.029, 16.283), with a default spacing of 0.375 Å. The ’ligand pool’ refers to the metabolites identified in the ethanolic extract via GC-MS (Table 4), which were selected based on their statistical significance in the volcano plot (Figure 3). The ligand structures were extracted from Pubchem (https://pubchem.ncbi.nlm.nih.gov/, accessed on 10 November 2024) [63]. The ligand structure format was converted to a PDB file using OpenBabel v2.4.1 software [64]. Ligand energy minimization was performed using the MMFF94 force field within Avogadro v1.2.0 (https://avogadro.cc/, accessed on 10 November 2024) [65]. Molecular docking was performed using AutoDock Vina version 1.1.2 [66]. The docking result was expressed as binding energy (kcal/mol). Receptor–ligand interactions were created using Discovery Studio version v24.1.0.23298 (BIOVIA, San Diego, CA, USA) [67].

## Figures and Tables

**Table 1:** Qualitative phytochemical analysis of ethanolic extract of *P. heleniae*.

Specialized Metabolites	Ethanolic Extract
Alkaloids	− *
Terpenoids	++ *
Taninns	+++ *
Quinones	++ *
Phenolic compounds	+++ *
Flavonoids	++ *

* − non-detected, ++ positive, +++ strongly positive.

**Table 2:** Quantitative phytochemical analysis of ethanolic extract of *P. heleniae*.

Specialized Metabolites	Content (μg/mg Extract)	Limit of Quantification (LOQ)
Total phenolics (μg GAE/mg extract)	574.7± 0.9 *	3.2
Total flavonoids (μg QE/mg extract)	2.5 ± 0.2 *	0.3

* Values are mean ± SD (*n* = 3).

**Table 3:** Antioxidant activity of ethanolic extract of *P. heleniae*.

TEAC (mg/gdw) *		
Sample	ABTS	DPPH
Ethanolic extract	1.03 ± 0.02	0.67 ± 0.06

* TEAC: Trolox equivalent antioxidant capacity.

**Table 4:** Metabolites identified by GC-MS in *P. heleniae*.

IL *	RT (min) *	Score *	LTPRI Exp *	LTPRI Lit *	Identified Metabolite Name	Super Class
2	2.785	84.5	1084.56	1083	1-Heptanol	Lipids and lipid-like molecules
2	2.941	72.8	1093.84	1077.25	1,2 Propanediol	Organic oxygen compounds
2	3.48	98.1	1125.93	1121.68	Cyclohexanol	Organic oxygen compounds
2	4.339	85.7	1177.11	1176	Propiophenone	Organic oxygen compounds
2	5.205	76.4	1228.69	1266	3,7-dimethyltropolone	Terpenoids
2	5.269	82	1232.52	1230	4,5-Dimethylundecane	Hydrocarbons
2	7.074	75.2	1335.86	1312	Methyl 3-hydroxyhexadecanoate	Lipids and lipid-like molecules
2	7.78	71.1	1373.57	1365	Gamma-nonalactone	Organoheterocyclic compounds
2	8.25	96.2	1398.66	1405	2,3,5-Trimethyldecane	Hydrocarbons
2	11.482	79.5	1571.38	1555	3-Hydroxy-4-methylbenzoic acid	Alkaloids and derivatives
3	11.722	84.6	1584.42	1351	alpha-Cubebene	Lipids and lipid-like molecules
3	13.295	80.9	1672.35	1771	1-Pentadecanol	Lipids and lipid-like molecules
2	13.301	96.5	1672.67	1667	5-Azulenemethanol, 1,2,3,3a,4,5,6,7-octahydro-.alpha.,.alpha.,3,8-tetramethyl-,[3S-(3.alpha.,3a.beta.,5.alpha.)]-	Terpenoids
3	13.369	78	1676.51	1569	Lauric acid	Fatty Acids
2	13.743	74.5	1697.58	1688	Alpha-selinene	Lipids and lipid-like molecules
2	14.319	97.7	1731.55	1727	5-Azulenemethanol, 1,2,3,4,5,6,7,8-octahydro-.alpha.,.alpha.,3,8-tetramethyl-, acetate, [3S-(3.alpha.,5.alpha.,8.alpha.)]-	Terpenoids
2	14.895	90.6	1765.58	1756.38	Trans-Nerolidyl formate	Organic acids and derivatives
3	24.105	85.6	2354.59	2192	Phytane	Lipids and lipid-like molecules
2	17.575	72	1931.45	1953	5-Octyl-2-pyridinecarboxylic acid	Alkaloids and derivatives
2	17.973	89.1	1957.26	1967	Hexadecanoic acid	Alkaloids and derivatives
2	18.618	96.4	1999.06	2005	Eicosane	Hydrocarbons
3	18.697	70.2	2004.42	1688	Protocatechuic acid methyl ester	Phenolic compounds
3	19.226	72.2	2040.46	1844	3-Methylene-7,11,15-Trimethyl-1-Hexadecene	Lipids and lipid-like molecules
3	20.079	82.8	2098.55	1786	Undecyl benzoate	Benzenoids
3	20.174	83.7	2105.28	1735	Benzoic acid, 2-ethylhexyl ester	Benzenoids
2	20.465	74.1	2125.89	2128	Methyl octadecanoate	Lipids and lipid-like molecules
2	20.928	81.5	2158.73	2172	Octadecanoic acid	Alkaloids and derivatives
2	21.423	99.9	2193.82	2195	Octadecanoic acid, ethyl ester	Alkaloids and derivatives
2	21.49	87.5	2198.62	2210	2,6,10,14,18-Pentamethyleicosane	Organic compounds
3	22.33	78.8	2248.85	2546	Erucic acid	Alkaloids and derivatives
2	22.641	92.9	2267.37	2275	Muscalure	Lipids and lipid-like molecules
3	23.057	77.8	2292.17	1950	Isophytol	Lipids and lipid-like molecules
3	23.287	82.8	2305.84	1980	Trans-9, Trans-12-Octadecadienoic Acid Methyl Ester	Lipids and lipid-like molecules
2	22.375	95.5	2311.13	2305	(11Z,14Z,17Z)-Methyl icosa-11,14,17-trienoate	Lipids and lipid-like molecules
2	23.637	96.5	2326.7	2321.57	Stearic acid	Lipids and lipid-like molecules
3	23.754	75.1	2333.7	2172	(Z)-9-Octadecenoic Acid Methyl Ester	Lipids and lipid-like molecules
3	23.774	81	2334.89	2101	(Z,Z,Z)-9,12,15-Octadecatrienoic Acid Methyl Ester	Lipids and lipid-like molecules
3	24.105	85.6	2354.59	2192	Hexadeca-2,6,10,14-tetraen-1-ol, 3,7,11,16-tetramethyl	Lipids and lipid-like molecules
2	24.143	77.4	2356.88	2371	tetracosane	Hydrocarbons
2	27.67	88.8	2566.96	2626.26	1,3-Propanediol, eicosyl ethyl ether	Organic oxygen compounds
2	29.826	97.3	2695.39	2700	9,12-Octadecadienoic acid (Z,Z)-, 2,3-dihydroxypropyl ester	Lipids and lipid-like molecules
3	29.855	74.9	2697.12	2697	Fumaric acid, dec-4-enyl hexadecyl ester	Organic acids and derivatives
3	30.288	70.3	2722.89	2500	13-Docosenoic Acid Methyl Ester	Lipids and lipid-like molecules
3	31.922	71.6	2820.23	2626	Docosyl isobutyl ether	Organic oxygen compounds
2	34.496	88.5	2973.58	2984	6-Methoxy-2,7,8-trimethyl-2-(4,8,12-trimethyltridecyl)chroman	Phenolic compounds
3	34.974	73.7	3001.07	3125	Desmosterol	Steroids and steroid derivatives
3	36.308	76.1	3081.54	3248	Stigmasterol	Steroids and steroid derivatives
3	37.444	70.7	3149.18	3343	Isofucosterol	Steroids and steroid derivatives

* IL, identification level according to [32]; RT, retention time in minutes; LTPRI Exp, experimental Van den Dool and Kratz retention index; LTPRI Lit, literature Van den Dool and Kratz retention index.

**Table 5:** Molecular docking analysis of main compounds with catalase protein.

Code	Ligand	Pubchem ID	Binding Energy (kcal/mol)	Ki (µM)
A	Stigmasterol	5280794	−11.0	8.58 nM
B	Desmosterol	439577	−8.3	0.82 µM
C	3,7-dimethyltropolone	13403206	−7.0	7.36 µM
D	3-Hydroxy-4-methylbenzoic acid	68512	−6.9	8.7 µM
E	Isophytol	10453	−6.5	17.11 µM
F	Protocatechuic acid methyl ester	287064	−6	39.8 µM

## Data Availability

The original contributions presented in this manuscript are publicly available.

## Supplementary Materials

The following supporting information can be downloaded at: https://www.mdpi.com/article/10.3390/molecules30061366/s1, Figure S1: Comparison of EC50 for ABTS assay. (Half Maximal Effective Concentration) values for antioxidant activity: Sample (A) vs. Trolox Standard (B); Figure S2. Comparison of EC50 for DPPH assay. (Half Maximal Effective Concentration) values for antioxidant activity: Sample (A) vs. Trolox Standard (B); Figure S3. Structural overlay of *Proteus mirabilis* catalase (2CAG, in red) and human catalase (8HID, in blue); Figure S4. Identification of regions of structural divergence between 2CAG and 8HID. Regions of structural divergence are highlighted with yellow spheres. The active site, including the heme group (green), is highly conserved between the two enzymes, showing differences mainly in peripheral regions.
