# Supplementary material for: Volatile Compositional Profile, Antioxidant Properties, and Molecular Docking of Ethanolic Extracts from Philodendron heleniae
Source: Molecules. 2025 Mar 18;30(6):1366. doi: 10.3390/molecules30061366 (PMC11946216; doi:10.3390/molecules30061366)
Supplement: Supplementary file 1 [file molecules-30-01366-s001.zip › molecules-3475652-supplementary.pdf]

Supplementary Materials

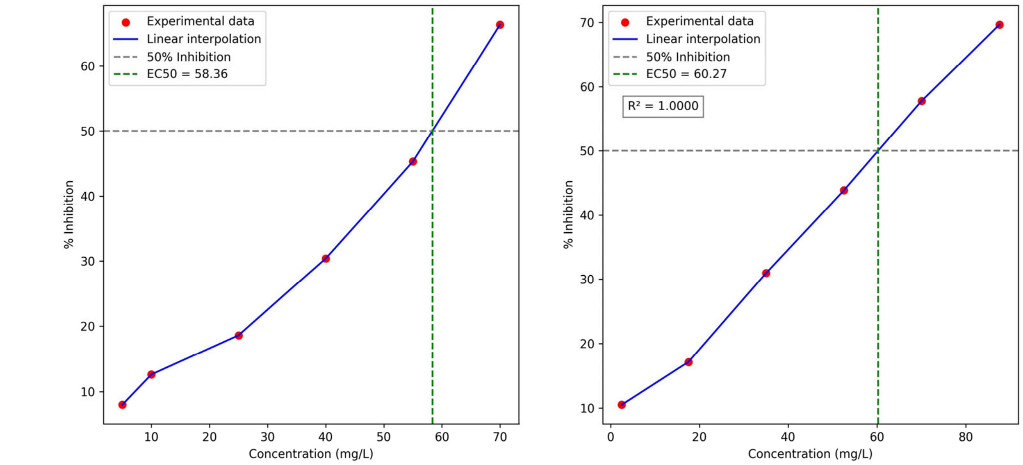

A **B**

Figure S1. Comparison of EC50 for ABTS assay. (Half Maximal Effective Concentration) values for antioxidant activity: Sample (A) vs. Trolox Standard (B).

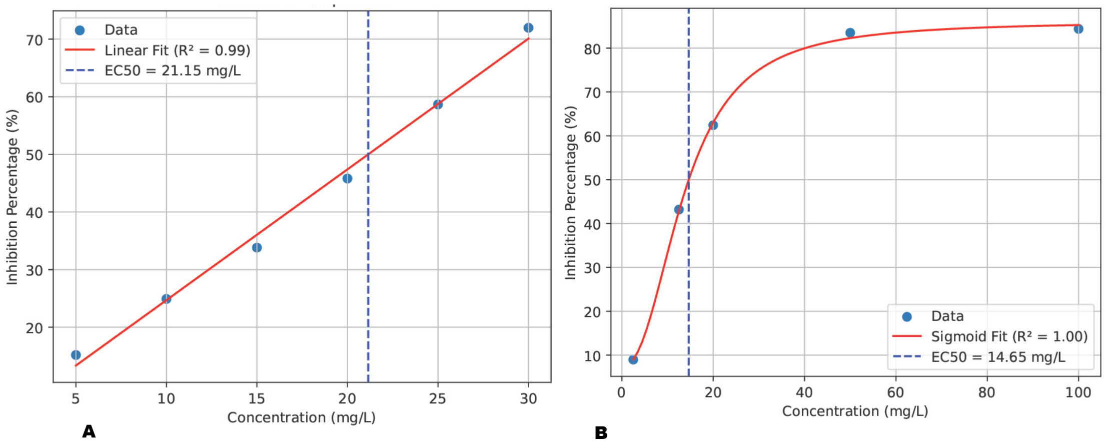

A **B**

Figure S2. Comparison of EC50 for DPPH assay. (Half Maximal Effective Concentration) values for antioxidant activity: Sample (A) vs. Trolox Standard (B).

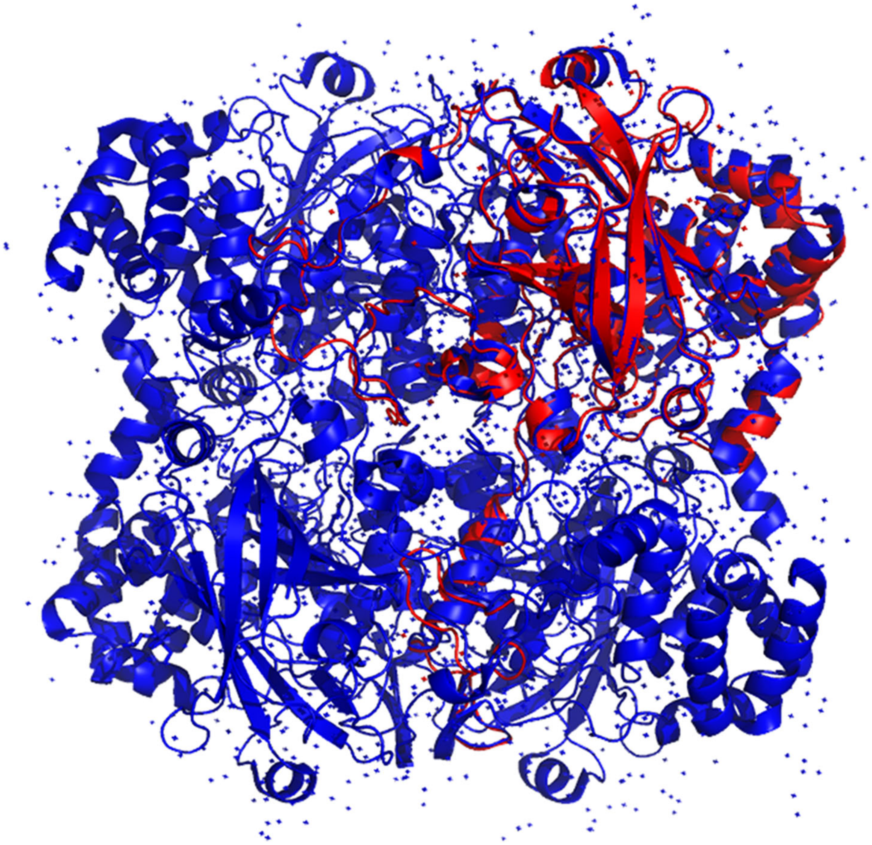

Figure S3. Structural overlay of *Proteus mirabilis* catalase (2CAG, in red) and human catalase (8HID, in blue).

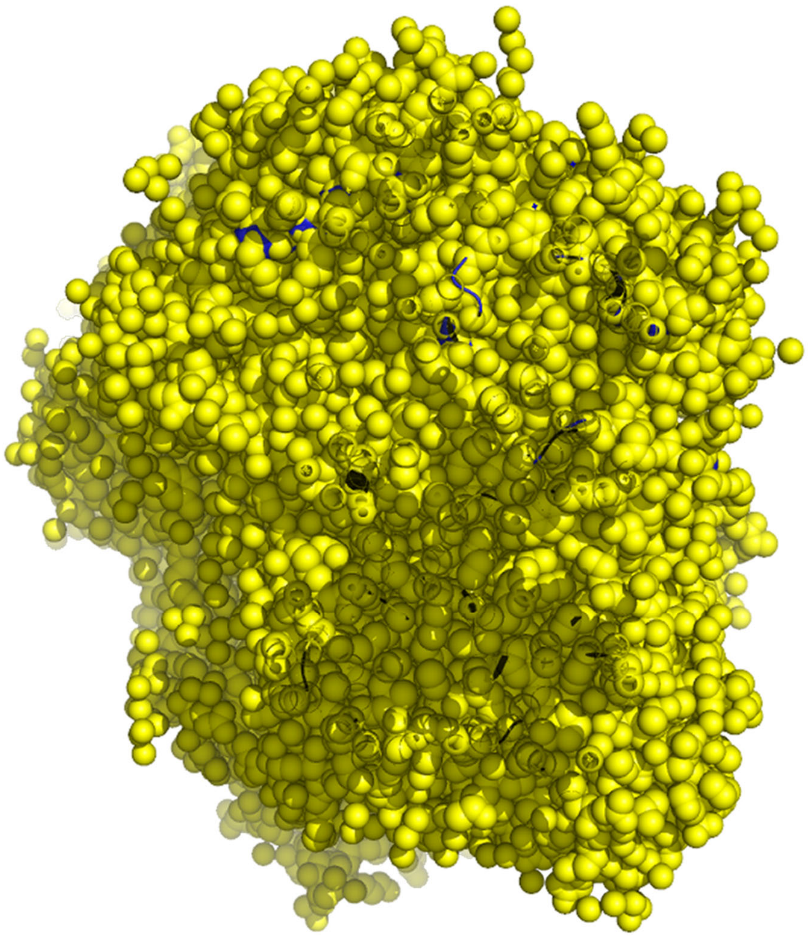

Figure S4. Identification of regions of structural divergence between 2CAG and 8HID. Regions of structural divergence are highlighted with yellow spheres. The active site, including the heme group (green), is highly conserved between the two enzymes, showing differences mainly in peripheral regions.
